# Ectopic sphenoidal ACTH-secreting adenoma revealed by ^11^C Methionine PET scan: case report

**DOI:** 10.1186/s12902-023-01298-2

**Published:** 2023-02-16

**Authors:** F. Lurquin, S. M. Constantinescu, R. M. Furnica, T. Duprez, C. Raftopoulos, L. Daoud, M. Lammens, D. Maiter

**Affiliations:** 1 Endocrinology and Nutrition Department, Cliniques Universitaires Saint-Luc, n°10, Avenue Hippocrate, 1200 Brussels, Belgium; 2 Department of Radiology, Cliniques Universitaires Saint Luc, UCLouvain, 1200 Brussels, Belgium; 3 Department of Neurosurgery, Cliniques Universitaires Saint Luc, UCLouvain, 1200 Brussels, Belgium; 4 Department of Pathology, Cliniques Universitaires Saint Luc, UCLouvain, 1200 Brussels, Belgium; 5 Department of Pathology, Antwerp University Hospital, University of Antwerp, 2650 Antwerp, Belgium

**Keywords:** Cushing’s syndrome, ACTH-secreting adenoma, Ectopic pituitary adenoma, ^11^C Methionine PET/MRI

## Abstract

**Background:**

Ectopic ACTH pituitary adenomas (EAPA), located outside the sella turcica and deriving from cellular remnants of Rathke’s pouch are a very rare cause of Cushing’s syndrome (CS). The diagnosis is often difficult and delayed, even after comprehensive work-up. To our knowledge, we report for the first time an ectopic corticotroph tumor of the posterior wall of the sphenoid sinus, leading to false positive results of bilateral inferior petrosal sinus sampling (BIPPS) and which was finally localized by a co-registered^11^ C Methionine PET/MR imaging.

**Case presentation:**

A 48-year-old woman was referred for a high clinical suspicion of ACTH-dependent CS. Biological testing comprising low dose dexamethasone suppression and CRH stimulation tests were indicative of pituitary Cushing’s disease, but comprehensive pituitary MRI did not reveal any pituitary adenoma. BIPSS confirmed however a central origin of ACTH secretion (central-to-peripheral ACTH ratio > 100) and revealed a significant right-to-left gradient (6.2), leading to a first right-sided exploratory hypophysectomy, that did not cure the patient. BIPSS images were reviewed and revealed preferential drainage of the left pituitary to the right petrosal sinus, leading us to a left sided exploratory hypophysectomy, which was again unsuccessful. A^11^ C Methionine PET/MRI was performed and revealed a hypermetabolic lesion adjacent to the posterior wall of the sphenoidal sinus. After surgical resection, this polypoid mass was identified as an ectopic ATCH-secreting pituitary adenoma expressing ACTH and T-Pit and complete remission of hypercortisolism was observed.

**Conclusions:**

In conclusion, we report a case of ACTH-dependent Cushing’s syndrome, caused by an ectopic corticotroph adenoma located in the sphenoidal sinus, which perfectly mimicked the biological features of a classical pituitary ACTH adenoma on a comprehensive hormonal evaluation including BIPPS, and the features of a benign naso-sinusal polyp at MRI. We report for the first time a key role of^11^ C Methionine PET co-registered to high resolution MRI for localizing ectopic adenomas, efficiently guiding surgical removal and leading to complete remission of hypercortisolism.

## Background

Ectopic ACTH pituitary adenomas (EAPA), located outside the sella turcica and on the embryological migration path of Rathke’s pouch (eg nasopharynx, clivus, sphenoidal sinus, cavernous sinus, suprasellar region) are a very rare cause of Cushing’s syndrome (CS) [1]. The diagnosis is often difficult and delayed, even after comprehensive work-up. To our knowledge, we report for the first time an ectopic corticotroph tumor of the posterior wall of the sphenoid sinus, leading to false positive results of bilateral inferior petrosal sinus sampling (BIPPS) and which was finally localized by a co-registered^11^ C Methionine PET/MR imaging.

## Case presentation

A 48-year-old woman was referred to our Endocrinology Department for suspicion of Cushing’s syndrome (CS). She complained of weight gain (7 kg in 1 year), face swelling and frequent bruising. Menstruations had been irregular for the last year. She also had recently suffered from a spontaneous rib fracture and aseptic osteonecrosis of the shoulder. Clinical examination revealed truncal adiposity (BMI 27.5 kg/m^2^) with buffalo neck, typical skin frailty, peripheral amyotrophy and hypertension (150/90 mmHg).

Biological work-up at admission, including blood count, hepatic enzymes, kidney function and electrolytes, was normal, except for elevated LDH at 435 U/l (normal values 140–280) and neutrophilia at 11,390/μl (normal values < 10,000). Hba1c was normal at 5.6%. A recent bone density scan had revealed osteoporosis at the femoral site (T-score − 3.1) and osteopenia in the lumbar spine (T-score − 1.9).

Morning cortisol and ACTH concentrations were high (Table 1), as was 24 h free urinary cortisol on three different collections (86.5, 99.3 and 131.0 μg/24 h, normal values < 40). The short 1 mg dexamethasone test and Liddle test (dexamethasone 0.5 mg every 6 hours for 48 hours) were both indicative of CS (Table 1). A corticotropin-releasing hormone stimulation test (CRH, 100 μg iv) induced a 57% elevation in ACTH (99 to 145 ng/l) and a 34% rise in cortisol (702 to 946 nmol/l), in favor of the diagnosis of Cushing’s disease (CD), while a desmopressin test (10 μg iv) remained inconclusive by yielding only a 15% increase in ACTH levels (103 to 119 ng/l) and an 18% rise in cortisol (726 to 856 nmol/l). The other pituitary hormones levels were normal, while DHEA sulfate and total testosterone were elevated (Table 1).

**Table 1:** Endocrinological work-up at diagnosis

Parameter (Units)	Results	Normal range
Morning cortisol (nmol/l)	669	130–500
Morning ACTH (ng/l)	89	5–49
TSH (mU/l)	0.78	0.27–4.2
Free T4 (pmol/l)	16.8	12–22
IGF-1 (μg/l)	148	102–267
LH (U/l)	3.9	1–95
FSH (U/l)	11.9	1.7–134
Oestradiol (ng/l)	23	12–398
Total testosterone (nmol/l)	2.94	0.27–1.7
DHEA-Sulfate (μmol/l)	14.4	1–7
Prolactin (μg/l)	12	5–23
24 h urinary free cortisol on three different collections (μg/24 h)	86.0, 99.3 and 131.0	< 40
Morning cortisol after 1 mg DXM (nmol/l)	369	< 50
Morning cortisol after Liddle test (nmol/l)	911	< 50

The Liddle test was performed by giving 0.5 mg dexamethasone every 6 hours for 48 hours

*ACTH* adrenocorticotropic hormone, *TSH* thyroid-stimulating hormone, *IGF-1* insulin-like growth factor 1, *LH* luteinizing hormone, *FSH* follicle-stimulating hormone, *DHEA* Dehydroepiandrosterone, *DXM* dexamethasone

**Fig. 1:**
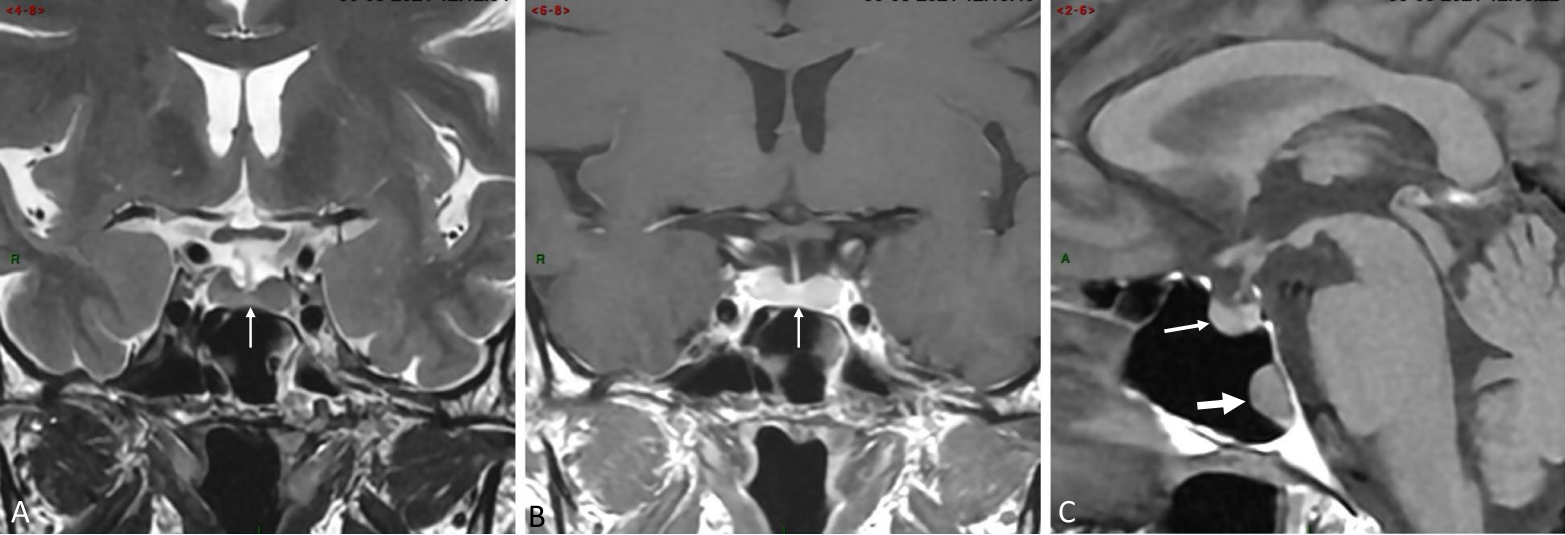
Pituitary magnetic resonance imaging (MRI) at diagnosis. **A** Thin coronal T2-weighted slice showing a normal pituitary gland (arrow); **B** Contrast-enhanced (CE) thin coronal T1-weighted slice in similar location as Fig. 1A, failing to reveal any abnormality in the pituitary gland; **C** Pre-contrast thin mid-sagittal T1-weighted slice showing normal anterior and posterior pituitary lobes (thin arrow) and a polypoid-like mass on the posterior wall of the sphenoid sinus, clearly distant from the sellar region and first considered as a benign naso-sinusal polyp (thick arrow)

Pituitary magnetic resonance imaging (MRI) including high resolution T2-weighted sequences, as well as pre- and post-contrast T1-weighted sequences with rapid dynamic acquisition failed to reveal an adenoma (Fig. 1A-C). Bilateral inferior petrosal sinus sampling (BIPSS) was therefore performed. A marked central-to-peripheral gradient in ACTH secretion was observed in both petrosal sinuses, suggesting the pituitary origin of ACTH hypersecretion (Table 2). Higher ACTH levels were recorded on the right side, leading to a significant lateralization ratio of 6.2.

**Table 2:** ACTH concentrations (in ng/l) recorded during bilateral inferior petrosal sinus sampling (BIPPS) at baseline and after intravenous injection of 0.100 mg of corticotropin-releasing hormone (CRH) at 0 minutes

Time	PV	RPS	LPS	RPS/PV	LPS/PV
- 5 min	46	941	151	20,6	3,3
0 min	45	1158	237	25,5	5,2
2 min	55	5939	1624	107,8	29,5
5 min	73	2275	865	31,3	11,9
10 min	79	3418	1150	43,3	14,6
15 min	81	3670	1029	45,5	12,8

*ACTH* adrenocorticotropic hormone, *CRH* corticotropin releasing hormone, *PV* peripheral vein, *RPS* right petrosal sinus, *LPS* left petrosal sinus

The patient underwent subsequently a first exploratory transsphenoidal surgery targeting the right side of the pituitary, with excision of a more friable part of the gland. Histology showed normal pituitary tissue, and postoperative cortisol levels remained elevated (681 nmol/l at day 2). Pituitary function was otherwise fully preserved.

Because of the lack of biological remission, a retrospective look on BIPSS images was done which highlighted a preferential cross drainage of the left pituitary towards the right petrosal sinus. The patient therefore underwent a second exploratory surgery now targeting the left side of the pituitary, with removal of a more soft part of the left pituitary. Again, post-operative cortisol level remained elevated at 543 nmol/l at day 2 and histopathological examination showed normal pituitary tissue. Importantly, pituitary function was again preserved without any hormonal deficit. A treatment with ketoconazole was initiated but rapidly discontinued because of nausea and abdominal pain. Other medical treatments such as metyrapone or osilodrostat were considered at this stage but the cost was prohibitive as they are not reimbursed by the Belgian social security.

**Fig. 2:**
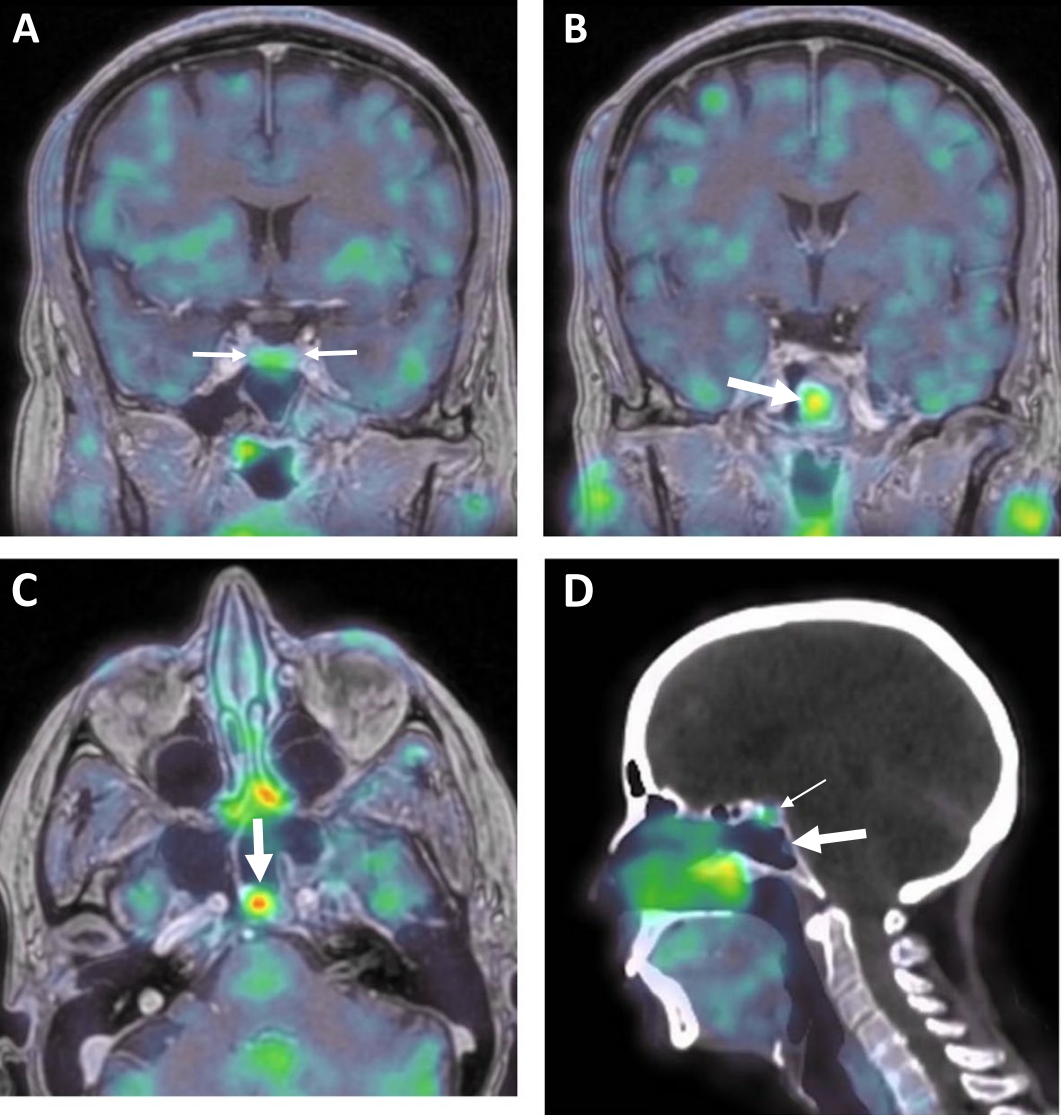
Co-registered magnetic resonance imaging (MRI) and ^11^C-methionine positron emission tomography (PET) performed after two unsuccessful pituitary surgeries. **A** Coronal view showing normal and homogeneous tracer uptake within residual pituitary parenchyma (between arrows); **B** Coronal view on a slightly more posterior view than Fig. 2A showing intense tracer uptake within the posterior sphenoid mass (large arrow), SUV_max_ at 3.3; **C** Axial transverse view showing similar features as Fig. 2B. **D** ^68^Ga-Dotatate-PET/CT showing normal uptake at the pituitary level (thin arrow) and no uptake of the tracer in the posterior sphenoid mass (large arrow)

Because of the lack of clinical and biological remission, further investigations were performed. A full body ^68^Ga-Dotatate-positron emission tomography (PET)/CT imaging failed to reveal a potential source of ectopic ACTH or CRH secretion. A ^11^C Methionine PET co-registered with high resolution 3D MRI was performed next and did not demonstrate a residual pituitary adenoma. This imaging procedure showed a normal uptake in the remaining pituitary gland (Fig. 2A), but revealed a hypermetabolic 9 × 6 × 8 mm mass (maximal standardized uptake value or SUV_max_ at 3.3) located on the posterior wall of the sphenoid sinus (Fig. 2B and C). Of note, this small polypoid process was already present on previous MR examination (Fig. 1C) and was negative on the ^68^Ga-Dotatate-PET (Fig. 2D). As it presented with similar MR features as those commonly seen for benign mucosal polyps and was distant from the sella, it had not been considered as a potentially ectopic adenoma.

**Fig. 3:**
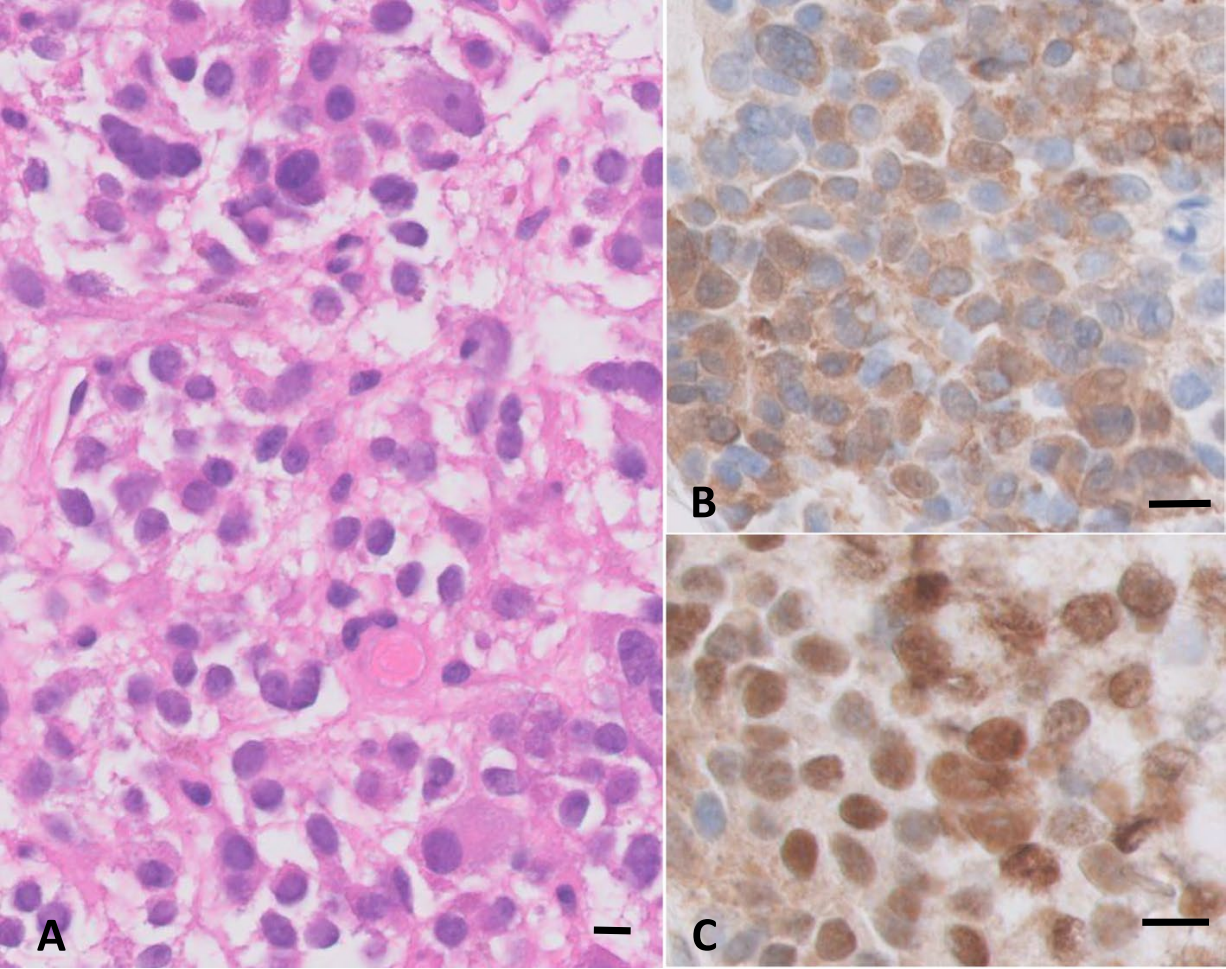
Histological and immunohistochemical analysis of the sphenoidal mass after surgical excision. **A** Hematoxylin and eosin staining of a tumor section, showing cells with sparsely granulated slightly basophilic cytoplasm. **B** Immunohistochemistry performed for ACTH, showing diffuse intra-cytoplasmic ACTH staining in the majority of tumoral cells. **C** Immunohistochemistry performed for T-Pit, revealing nuclear staining of the tumor cells. (Bar = 10 μm)

A third trans-sphenoidal surgical procedure was performed by the same neurosurgeon (CR) to remove this posterior sphenoidal mass. Serum cortisol decreased to very low levels (30 nmol/l) on post-operative day 2 and the patient was started on hydrocortisone replacement therapy. Histological examination confirmed the presence of pituitary adenomatous tissue with a Ki67 proliferation index at 1–2% and positive immunohistochemical staining for both ACTH and T-Pit (Fig. 3A-C). During follow-up, clinical signs of hypercortisolism rapidly improved and severe corticotropic insufficiency persisted 2 months after surgery (low morning cortisol at 2.7 nmol/l), confirming remission of CS.

## Discussion

We report a challenging case of severe ACTH-dependent Cushing’s syndrome without any identifiable pituitary tumor despite adequate MR imaging and two exploratory transsphenoidal surgeries, while preoperative hormonal testing and bilateral inferior petrosal sinus sampling (BIPSS) were highly consistent with a pituitary origin of ACTH secretion. We highlight the essential role played by a co-registered ^11^C Methionine PET/MRI imaging procedure in the diagnostic process, as it allowed to suspect an ectopic ACTH secreting adenoma deeply located on the posterior wall of the sphenoid sinus, mimicking a benign naso-sinusal mucosal polyp. The diagnosis was confirmed by post-surgical remission of CS and identification of pituitary corticotroph cell lineage of the resected tumor which showed positive ACTH and T-Pit expression.

A recent review highlighted that ACTH-secreting adenomas are the most frequent ectopic pituitary adenomas [1]. They can be located in the sphenoid sinus, the suprasellar region, the cavernous sinuses or in other locations. The diagnosis of such lesions is often difficult and delayed, even after comprehensive work-up including BIPSS.

Bilateral inferior petrosal sinus sampling (BIPSS) is indicated to distinguish ectopic ACTH secretion from Cushing’s disease, particularly in cases of negative MR imaging in patients with biological evidence of ACTH-dependent cortisol excess [2]. False negatives or false positives may however occur when the purpose of performing BIPSS is to define the exact location of the ACTH source. Anatomical variants of the petrosal venous system have been described, which potentially result in abnormal venous drainage. For instance, the petrosal sinus can drain immediately into the jugular or the vertebral vein [3]. Conversely, some extra pituitary tumors can mimic pituitary location with preferential lateralized secretion, notably in patients with ectopic CRH secretion [4] or in the presence of ectopic pituitary adenoma within the sphenoid sinus as it was observed in our case.

In our patient, in spite of negative pituitary MR examination, the very high central/peripheral ACTH gradient at BIPSS (above 100) convinced us to perform exploratory pituitary surgery, initially targeted to the right side and thereafter to the left side, as advocated by recent surgical guidelines [5]. We now hypothesize that the tumor, located medially and posteriorly in the sphenoid sinus, preferentially drained into in the right petrosal sinus. We also encountered another pitfall in our diagnostic approach, as BIPPS highlighted a marked right-sided gradient, which was a false positive. We discovered later, after the first surgical exploration, important contralateral cross-drainage, which triggered the decision of a second surgical intervention targeting the left pituitary lobe. To our knowledge, major preferential drainage in the opposite petrosal sinus is rare, but still more frequent than extra-pituitary ACTH producing tumors.

MRI has a stronger positive predictive value than BIPSS to determine pituitary tumor lateralization and current guidelines therefore recommend to perform BIPSS only in case of negative MRI to lateralize the lesion [6]. However, in case of a negative first BIPPS-guided surgical exploration, a surgical exploration of the entire pituitary gland is recommended before performing extended hemi-hypophysectomy, because of the limited accuracy of BIPSS. In a recent series, as much as 31% of ACTH-secreting adenomas were located contra laterally to the side predicted by BIPSS [6].

To our knowledge, we report for the first time localization of such an ectopic corticotroph tumor by co-registered ^11^C Methionine PET/MRI. As methionine is an amino-acid used for protein synthesis, ^11^C Methionine is preferentially taken up by protein secreting adenomas and has shown a sensitivity of 70–100% in recent studies involving Cushing’s disease patients [7, 8]. Recent studies have shown that nuclear imaging techniques may provide consistent results in localizing pituitary adenomas when MRI alone failed to locate them [7, 8], and ^11^C Methionine PET/MRI is considered superior to ^18^FDG-PET/MRI or CT for this purpose [9]. Indeed, ^11^C Methionine PET/MRI has demonstrated encouraging results in this indication [10], and seems to offer greater accuracy and positive predictive value than BIPSS [10]. Nuclear imaging could gain more importance in the decision-making process in ACTH dependent Cushing’s syndrome and could even identify, or at least suspect, ectopic pituitary adenomas, as illustrated by this case report.

Ectopic pituitary adenomas originate from cellular remnants of the migration of Rathke’s pouch cells along the pharyngeal tract and have been shown to remain under the regulatory control of the hypothalamus [11, 12]. In our case, dynamic testing was compatible with an ACTH-producing pituitary adenoma, as CRH stimulation induced a significant increase in ACTH and cortisol. We also show for the first time that such ectopic pituitary adenomas also express T-Pit, a transcription factor specifically expressed in pituitary cells that acquire a corticotroph phenotype [13].

In conclusion, we report a case of ACTH-dependent Cushing’s syndrome, caused by an ectopic corticotroph adenoma located in the sphenoid sinus, which perfectly mimicked the features of a classical pituitary ACTH adenoma on a comprehensive hormonal evaluation including BIPPS, and the features of a benign naso-sinusal polyp at MRI. We report for the first time a key role of ^11^C Methionine PET co-registered to high resolution MRI for localizing ectopic adenomas, efficiently guiding surgical removal and leading to complete remission of hypercortisolism.

## Data Availability

All data presented in this paper is available from the corresponding author upon reasonable request.

## Abbreviations

ACTH: adrenocorticotropic hormone
BIPSS: bilateral inferior petrosal sinus sampling
CS: Cushing’s syndrome
MRI: magnetic resonance imaging
PET: positron emission tomography
